# Chemical Constituents, Enantiomer Content, Antioxidant and Anticholinesterase Activities of *Valeriana microphylla* Kunth Essential Oil

**DOI:** 10.3390/plants12112155

**Published:** 2023-05-30

**Authors:** Gabriela Aguilar, James Calva, Luis Cartuche, Melissa Salinas, Chabaco Armijos

**Affiliations:** Departamento de Química y Ciencias Exactas, Universidad Tecnica Particular de Loja, Loja 1101608, Ecuador

**Keywords:** *Valeriana microphylla*, essential oil, AChE, BuChE, antioxidant activity

## Abstract

The study of the essential oil (EO) from aerial parts (stems and leaves) of *Valeriana microphylla* Kunth (Valerianaceae), collected from the Saraguro community in the southern region of Ecuador, was analyzed for the first time. A total of 62 compounds were identified in *V. microphylla* EO by GC-FID and GC-MS on nonpolar DB-5ms and polar HP-INNOWax columns. The most abundant components (>5%) detected on DB-5ms and polar HP-INNOWax columns were *α*-gurjunene (11.98, 12.74%), germacrene D (11.47, 14.93%), *E*-caryophyllene (7.05, 7.78%), and *α*-copaene (6.76, 6.91%), respectively. In addition, the enantioselective analysis, carried out on a chiral column, showed (+)-α-pinene and (R)-(+)-germacrene as enantiomerically pure compounds (enantiomeric excess = 100%). The antioxidant activity was high for the radicals ABTS (SC50 = 41.82 µg/mL) and DPPH (SC50 = 89.60 µg/mL), and finally, the EO was shown to be inactive to the enzyme acetylcholinesterase (AChE) and butyrylcholinesterase (BuChE), as both values were >250 µg/mL.

## 1. Introduction

The Valerianaceae family is widely distributed and about 350 species are grown worldwide (except in countries of the oceanic continent, such as Australia and New Zealand), which are mainly distributed at high elevations and in alpine areas [1]. The *Valeriana* genus, belonging to the Valerianaceae family consisting of 250 species, is distributed in the Andes (South America), such as Peru, Ecuador, Colombia, Chile, and Argentina [2]. In Ecuador, this botanical family is represented by 35 species, and *Valeriana* is a typical genus of high and cold areas; seven species are endemic, and most are shrubby and grow in the Andean forests up to the sub-páramos: *Valeriana alypifolia* H.B.K., *V. buxifolia* F.G. Meyer, *V. hirtella* H.B.K., *V. imbricata* Killip, *V. microphylla* H.B.K., *V. protenta* Eriksen, and *V. punctata* F.G. Meyer [3,4].

**Figure 1:**
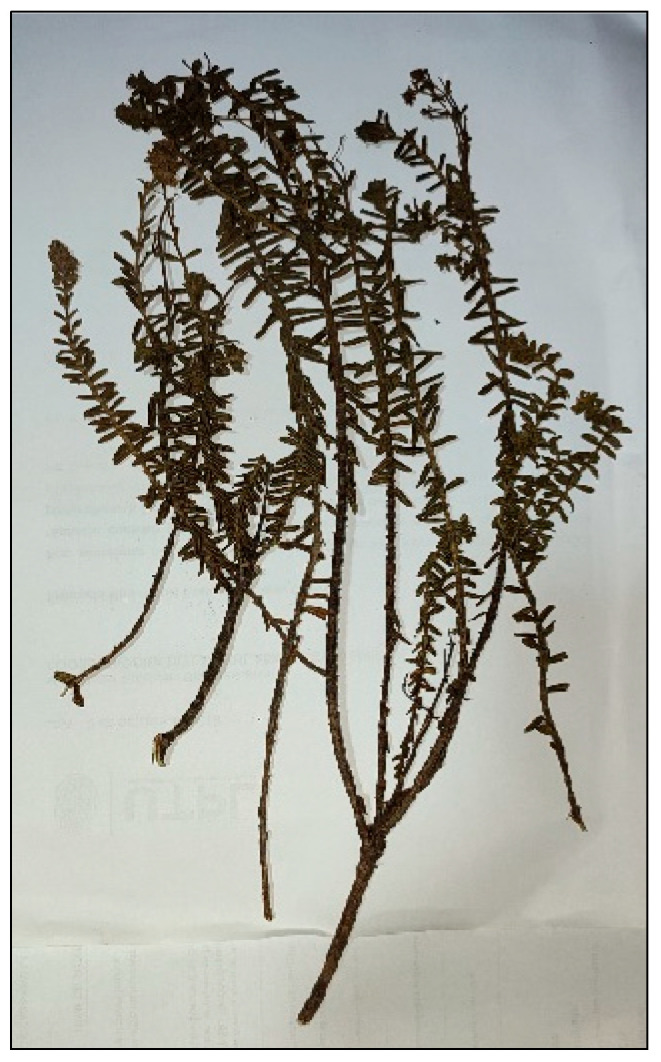
Image of *V. microphylla* Kunth collected in Ecuador.

*Valeriana microphylla* Kunth is commonly known as “*valeriana* de cerro”, “candelilla”, or “escobilla” by the Saraguros community of Ecuador, as well as “sacha *valeriana*” and “warmi *valeriana*” in Kichwa-Spanish language, and as “hapapu”, “hata”, or “yana yanta” in Kichwa [5]. Morphologically, they are small plants measuring 60 cm in length with a fetid odor, and the leaves are oval or elliptical and up to 10 mm long, as seen in Figure 1. They have inflorescence at the tips of the upright branches, up to 5 cm long, with many flowers; these can be white or pink, sometimes with lilac tints, up to 3 mm long, and tubular in shape. The fruit are crowned lunular in shape and measure up to 1.5 mm [6].

In Ecuador, *V. microphylla* is widely distributed in the Andean region from 2500 to 4500 m a.s.l. in the provinces of Imbabura, Bolívar, Cañar, Carchi, Chimborazo, Cotopaxi, Tungurahua, Morona Santiago, Napo, Pichincha, Sucumbíos, Azuay, Zamora Chinchipe, and Loja [7].

Traditional and ancestral uses for heart, nervous, and stomach conditions have been reported for *V. microphylla*. Other medicinal uses, such as antipyretic, antipruritic, diuretic, purgative, and antispasmodic, have also been registered [8]. The “Saraguros” indigenous community’s (southern Ecuador) knowledge of medicinal plants is broad, and they use this species to treat and cure diseases related to nerves [9]. The Valerian species and other genera belonging to the Valerianaceae family have used the roots and rhizomes in traditional medicine for centuries as a sedative, and many species have been studied so far [10]. The main product obtained from this species is the extract of roots, although some of its parts have various traditional uses; for example, the root bark is used as an infusion to cure fear, the root and the flower along with other herbs are used for the same purpose, and the leaves are used as an infusion to make baths against *hechizos*, *malos espíritus*, and *enfriamientos* [5].

Studies of the chemical composition of various Valerian taxa have reported the presence of flavonoids and alkaloids obtained mainly from the aerial parts, while sesquiterpenes and iridoids were found in the roots and rhizomes [11]. The most important iridoid compounds found in Valerianaceae were valepotriates, valtrate, isovaltrate, acevaltrate, and didrovaltrate, which were responsible for the sedative effect [10]. Other related studies of species belonging to the *Valeriana* genus showed a high level of antioxidant effects, such as in the essential oil (EO) of *Valeriana jatamansi* obtained from aerial parts and roots collected in the pre-flowering stage [12], while *Valeriana officinalis* has helped to block the production of reactive oxygen species in brain slices induced by quinolinic acid [13].

EOs are a miscellaneous family of low molecular weight organic compounds with circumstantial biological activity, being of interest for the control of human ailments, such as Alzheimer’s disease. Several in vitro analyses showed that some components in EOs may have cholinesterase (ChEs) inhibitory activity [14,15]. Acetylcholinesterase (AChE), together with butyrylcholinesterase (BuChE), belongs to the group of enzymes called cholinesterases, which catalyze the hydrolysis/degradation of the neurotransmitter acetylcholine (Ach), resulting in an increase in the level of ACh in the neuronal synaptic area. In a healthy normal brain, AChE predominates over BuChE activity [16,17]. Reports of in vitro ChEs enzymes inhibition activity of species of the Valerian genus showed that the sesquiterpene volvalerenal acid K, when isolated from *V. officinalis* var. *latiofolia*, increased learning and memory abilities in mice as their brain tissues were improved [18]. The evaluation of the anticholinesterase activity of *V. microphylla* will be reported for the first time in the present investigation.

Traditionally, the rural communities of southern Ecuador use the roots of the plant as a therapeutic resource, which can be macerated in *aguardiente* (alcohol), and the exudate of its roots can be obtained for long periods of time in dark or buried places, while the aerial part is discarded and used solely as fertilizer. Therefore, since there is little information in the scientific literature on the chemical composition of the aerial parts of valerian species that allow for the evaluation of its possible use [19], the purpose of this research is to provide information on the chemical composition and antioxidant and cholinesterase activity of the essential oil of the aerial parts (leaves and stems) of *V. microphylla* from Ecuador.

## 2. Results

### 2.1. Physical Properties

Steam distillation from *V. microphylla* leaves and stems provided an EO with an intense yellow color and characteristic aroma of the species, with an average yield of 0.0122 ± 0.0031% (*w*/*w*).

### 2.2. GC-EIMS and GC-FID Analyses

**Figure 2:**
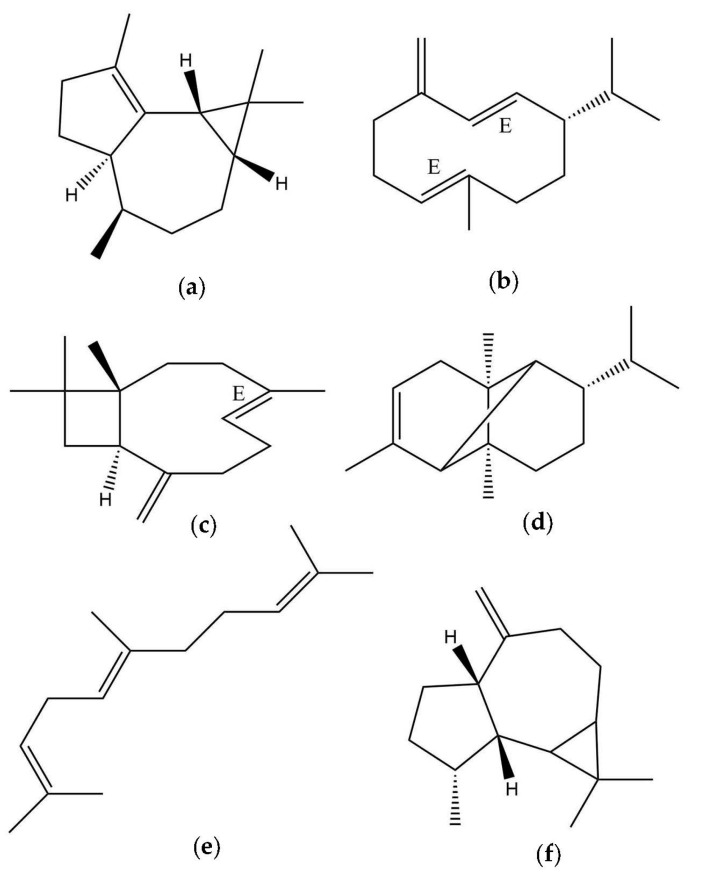
Principal compounds *V. microphylla* EO; (**a**) *α*-gurjunene; (**b**) germacrene D; (**c**) *E*-caryophyllene; (**d**) *α*-copaene; (**e**) *E,E-α*-farnesene; (**f**) allo-aromadendrene.

**Figure 3:**
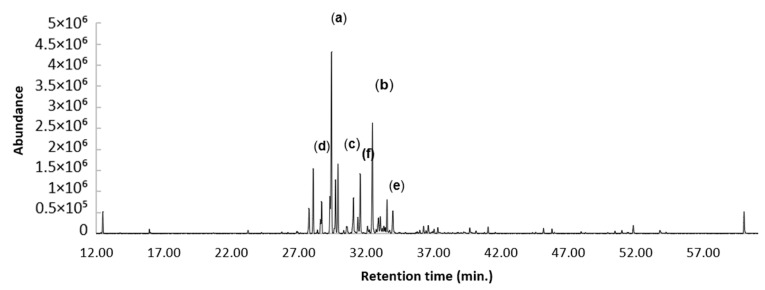
GC/EMS chromatogram of *V. microphylla* on a nonpolar DB5-ms column (a) *α*-gurjunene; (b) germacrene D; (c) *E*-caryophyllene; (d) *α*-copaene; (e) *E,E*-α-farnesene; (f) allo-aromadendrene.

A total of 62 constituents were identified and quantified in the essential oil of *V. microphylla* by GC/FID and GC/MS on nonpolar DB-5ms and polar HP-INNOWax columns. The major constituents (>4%) detected by the two columns were *α*-gurjunene (11.98, 12.74%) (a), germacrene D (11.47, 14.93%) (b), *E*-caryophyllene (7.05, 7.78%) (c), *α*-copaene (6.76, 6.91%) (d), *E,E*-α-farnesene (4.78, 4.13%) (e), and allo-aromadendrene (4.25, 2.55%) (f), respectively (Figure 2). All the identified constituents represented 98.08% and 98.29% of the total EO. This was calculated as the sum of the integrated peaks of the identified compounds, with respect to the total area of the peaks in the gas chromatograms. The compounds identified in the *V. microphylla* EO, with the corresponding percentages, are listed in Table 1. On the other hand, the GC-MS chromatogram is shown in Figure 3.

The main constituents were sesquiterpene hydrocarbons (75.15%, 79.31%), followed by aliphatic hydrocarbons (6.27%, 8.89%), oxygenated sesquiterpenes (5.07%, 3.49%), and other compounds (5.20%, 1.85%).

### 2.3. Enantioselective Analysis

Enantioselective analysis was performed by GC/MS of the essential oil and reported for first time, and the (+)-*α*-pinene and (+)-germacrene D were recognized as enantiomerically pure (enantiomeric excess = 100%). The enantiomeric distribution (%) for each compound are present in Table 2.

### 2.4. Cholinesterase Inhibition Test

The cholinesterase activity of *V. microphylla* EO was evaluated for the first time. It was shown to be inactive against the AChE and BuChE enzymes, both having values > 250 µg/mL, respectively. Donepezil hydrochloride was used as a positive control. The half maximum inhibitory concentration (IC_50_) values are presented in Table 3.

### 2.5. Antioxidant Activity

The antioxidant capacity of the EO of *V. microphylla* was expressed as the concentration needed for the radical scavenging capacity at 50% (SC_50_). According to the results depicted in Table 4, *V. microphylla* EO exerted a strong to moderate scavenging effect over ABTS cation radical and DPPH radical, with SC_50_ values less than 100 µg/mL.

## 3. Discussion

The average yield of aerial parts of *V. microphylla* EO obtained in relation to the plant material used was a low value of 0.0122 ± 0.0031% (*w*/*w*) [37]. There are factors that influence the variation in the yield of EOs, such as plant species, origin, part of the plant used (leaves, stems, flowers, roots, etc.), climatic and cultivation conditions (use of fertilizers, cultivation soil, temperature) [38], the extraction method (steam distillation and hydrodistillation), which has some weaknesses that greatly affect the yield and quality, and the way to store the EO [39,40].

The chemical composition of EO obtained from the aerial parts of *V. microphylla* species was reported for the first time, and a total of 62 compounds were identified. The major compounds were *α*-gurjunene (11.98, 12.74%), germacrene D (11.47, 14.93%), *E*-caryophyllene (7.05, 7.78%), *α*-copaene (6.76, 6.91%), *E*,*E*-α-farnesene (4.78, 4.13%), and allo-aromadendrene (4.25, 2.55%). The chemical profile of EO was compared with studies on EO samples obtained from the root of species from India, such as *V. jatamansi* and *V. hardwickii*, among which sesquiterpene (36.1%) was reported; α-gurjunene (8.7, 4.1%) and allo-aromadendrene (1.6, 0.3%), respectively, were reported [41]. In contrast, among the majority compounds of *V. officinalis* root, the EO included (*E*)-caryophyllene (3.4%), allo-aromadendrene (4.1%), and *α*-gurjunene (1.2%), and germacrene D (0.7%) is found in a minority within the composition [42]. The EO obtained by the hydrodistillation of underground parts of *V. alliariifolia* reported *α*-gurjune (0.2%) and *α*-copaene (0.4%) among its minority compounds [43].

For the first time, the EO of *V. microphylla* was subjected to enantioselective analysis. The enantiomeric composition of an EO is an important property of the oil and an essential marker for determining the authenticity of a plant species [44]. In our study, we identified the enantiomers of germacrene D, which is considered a precursor of many sesquiterpenes, and its enantiomers are synthesized by plants, fungi, and animals [45]. The (+)-germacrene D has been found in some species, catalyzed by two enantioselective synthases [46], and another enantiomer was (+)-α -pinene, which research showed has potential as an inhibitor of phospholipase and esterase activities [47].

The aerial parts of *V. microphylla* EO showed moderate antioxidant activity with SC_50_ 41.82 ± 1.62 µg/mL for ABTS and SC_50_ 89.60 ± 1.31 for DPPH. Thusso et al. found that the methanolic and aqueous extract of *V. jatamansi* showed antioxidant activity with SC_50_ 78 ± 2.9 µg/mL and SC_50_ 154 ± 4.6 µg/mL, respectively, while the essential oil of its roots showed poor radical scavenging activity (SC_50_ 876 ± 12.8 µg/mL) by DPPH assay [48]. The moderate antioxidant activity of the essential oil could be attributed to the presence of sesquiterpenes [49]. The study on *V. jatamansi* species showed a high antioxidant activity in EO samples of roots (6.30 ± 0.047 µg/mL DPPH) and methanolic extracts of aerial parts (6. 82 ± 0.10 µg/mL ABTS) collected at the pre-flowering stage, while it was low at flowering (aerial parts) (2.91 ± 0.01 µg/mL ABTS) (2.07 ± 0.08 µg/mL DPPH) and post-anthesis (root) (2.94 ± 0.18 µg/mL ABTS) (3.44 ± 0.17 µg/mL DPPH) [12]. Wang et al. found a DPPH value of 493.40 µg/mL in the essential oil obtained from the roots and rhizomes of *V. officinalis* [50].

Exposure to radiation triggers the accumulation of antioxidants in the aerial parts, while under normal conditions, it stimulates their storage in the rhizome [51]. In addition, Rather et al. in their study carried out the determination of the antioxidant activity of *E*-caryophyllene, germacrene-D, and *β*-pinene, which presented a synergistic effect, obtaining a moderate dose-dependent antioxidant result against DPPH with values of 78.1 µg/mL, 73.2 µg/mL, and 80.0 µg/mL, respectively [52]. Consequently, we could attribute the antioxidant effect of *V. microphylla* EO to the presence of these major compounds.

An in vitro study showed that some components present in EOs may inhibit ChEs, whose function is to hydrolyze acetylcholine in the cholinergic terminals, and therefore, in some dementias where this neurotransmitter is diminished, such as Alzheimer’s disease, it could be an alternative pathway for the control of this disease [53]. The essential oil of *V. microphylla* reported values >250 µg/mL for AChE and BuChE, respectively. Studies on chloroform and ethyl acetate extracts of *V. wallichii* significantly inhibit AChE (IC_50_ 0.061 mg/mL) and BChE (IC_50_ 0.058 mg/mL), while extracts of *V. polystachya* also show a slight inhibition of AChE at 200 µg/mL [54]. Reports of ChEs activities in traditional medicinal species from Vietnam [55] have shown that Eos with high concentrations of sesquiterpenes exhibit moderate AchE inhibitory activity. The inhibitory activity of Aes from other species of the genus *Valeriana* against ChEs has been poorly documented.

Based on these findings, we can conclude that most species belonging to the Valerianaceae family are used for their numerous medicinal properties; in particular, their roots and rhizomes are used to prepare infusions. The present study emphasizes the aerial part of the Valerian species since this part is disregarded; for that reason, our purpose is to highlight its importance by determining its chemical composition and its activity as a natural antioxidant, this being an area of growing interest, especially in complementary medicine and food science.

## 4. Materials and Methods

### 4.1. Plant Material 

The aerial parts (leaves and stems) of the species *V. microphylla* were collected in October 2020 in the Saraguro province of Loja, at coordinates 9,593,252 N, 696,030 E, at an altitude of 3210 m.s.n.m. The collection was permitted by the Ministry of Environment Ecuador (MAE) with authorization MAE-DBN-2016-048.The plant was identified by José M. Andrade, curator of the Herbarium (HUTPL) at the Universidad Técnica Particular de Loja. A voucher sample of *V. microphylla* (PPN-va-001) was deposited in HUTPL.

### 4.2. Extraction of the Volatile Oil

The EO of *V. microphylla* was obtained by the steam distillation of fresh aerial parts (stem and leaves) for 3 h at atmospheric pressure using a Clevenger-type apparatus. The distilled EO was separated from the aqueous phase and dried over anhydrous sodium sulfate, filtered, and then stored in a brown vial at 4 °C until chemical and biological analysis. The procedure was performed in triplicate [56].

### 4.3. Chemical Characterization of Essential Oil

#### 4.3.1. Qualitative and Quantitative Analysis

Gas chromatography/mass spectrometry (GC/MS) analyses were performed in an Agilent Technologies model 6890 N gas chromatograph (GC) coupled to a mass spectrometer detector (model Agilent 5973 inert) (Santa Clara, CA, USA). A solution (1 μL) of each EO in dichloromethane (1:100 *v*/*v*) was injected in duplicate in split mode (40:1) at 20 °C. The GC equipment operated in electron ionization mode at 70 eV with helium as the carrier gas (1.00 mL/min in constant flow), and the GC oven operated with a temperature ramp from 60 °C to 250 °C, with a gradient of 3 °C/min and the ion source at 250 °C. In addition, capillary columns DB-5ms fused silica column (5% phenyl 95% polydimethylsiloxane, 30 m × 0.25 mm i.d., film thickness 0.25 μm) and HP-INNOWax (polyethylene glycol, 30 m × 0.25 mm i.d., film thickness 0.25 μm) were both purchased from J & W Scientific (Folsom, CA, USA). The procedure was carried out in triplicate.

In order to identify the compounds, present in EO, the mass spectra and the linear retention index (LRI) were compared with those reported in the literature. The LRI was determined experimentally according to Van Den Dool and Krats [57]. A homologous series of C9 to C24 alkanes were injected under the same conditions.

Gas chromatography/Flame ionization detector (GC/FID) was used for the quantitative analysis of the EO of *V. microphylla*. The prepared samples were injected under the same conditions as the GC/MS method, using the same columns and analytical conditions. To determine the percentage of aromatic compounds, the GC peaks were compared with the total area of the identified peaks [58]. According to the methodology proposed by Gilardoni et al. [59], a calibration curve was established for each column using isopropyl caproate (0.6, 1.8, 4.3, 8.3, 16.8, and 34.3 mg isopropyl caproate in 10 mL cyclohexane) and n-nonane (7 mg) as the calibration and internal standards, respectively. The limit of detection (LOD) (0.4 μg/mL) and the limit of quantitation (LOQ) (1.2 μg/mL) were determined. The correlation between both calibration curves was 0.995.

#### 4.3.2. Enantioselective Analysis of Essential Oil 

Enantioselective analysis was carried out for the first time in the aerial parts of *V. microphylla* EO. Capillary chiral was a Megadex (2,3-diethyl-6-tert-butyldimethylsilyl-cyclodextrin) 25 m × 0.25 mm I.D. × 0.25 µm df (Mega, Legnano, Italy). Temperature program: 50–200 °C at 2 °C/min, split mode 1:20; gas carrier Helium, injected volume 1.0 µL, to determine the order of elution of the enantiomers in EO, enantiomerically pure standards were injected under the same conditions [60].

### 4.4. AChE and BuChE Inhibition Spectrophotometric Analysis 

The anti-AChE and anti-BuChE activities in vitro of the EO were evaluated according to Calva et al. [56] and Ellman et al. [61].

A typical 200 μL inhibition assay volume contained phosphate buffered saline solution (pH 7.4), DTNB (1.5 mM), and a tested sample in DMSO (1% *v*/*v* final). Both AChE (Type V–S, lyophilized powder, 744 U/mg solid, 1272 U/mg protein) and BuChE (lyophilized powder, 900 U/mg protein) were dissolved in PBS (pH 7.4) and used at 25 mU/mL for the assay. After 10 min of pre-incubation, the enzyme substrate acetylthiocholine iodide (1.5 mM) was added to start the reaction. During 30 min of incubation at 30 °C, 96-well microtiter multiplate were read on a PherastarFS (BMG Labtech, Ortenberg, Germany) detection system. Enzymatic activities were tested in the presence of 0.05–250 μg/mL of each EO dissolved in DMSO, whose concentration was kept constant and expressed versus DMSO alone. Donepezil was used a reference ChE inhibitor for both enzymes [17]. IC50 values were determined from three individual experiments. The activity results were expressed as the mean ± SD of the three replicates. IC_50_ values were determined from a nonlinear regression model by using the GNUPLOT online package (www.ic50.tk (accessed on 1 March 2023), www.gnuplot.info (accessed on 5 March 2023)). One-way ANOVA simple or multiple comparison as well as [58] were used.

### 4.5. Antioxidant Spectrophotometric Analysis 

#### 4.5.1. DPPH Assay 

The DPPH radical scavenging assay was performed with slight modifications using the free radical 2,2-diphenyl-1-picrylhydryl (DPPH-) according to the method proposed by Thaipong et al. [62]. A working solution was prepared by dissolving 24 mg of DPPH in 100 mL of methanol, which was then stabilized in an EPOCH 2 microplate reader (BIOTEK, Winooski, VT, USA) at 515 nm until reaching an absorbance of 1.1 ± 0.01. Different concentrations of EO (1, 0.5, and 0.25 mg/mL) were used for the anti-radical reaction between EO and the free radical. Subsequently, 30 μL of EO sample and 270 μL of DPPH-adjusted working solution were added to a 96-well plate. The reaction was monitored at 515 nm for 60 min at room temperature. The blank and positive controls were methanol and Trolox, respectively. The results were expressed as SC50 (radical scavenging concentration at 50%) and calculated according to the corresponding curve fit of the data with GraphPadPrism v.8.0.1. Three replicates were used to measure.

#### 4.5.2. ABTS Assay 

According to Arnao et al. [63] and Thaipong et al. [62], antioxidant capacity was measured against the ABTS-+ cation (2,20-azinobis-3-ethylbenzothiazoline-6-sulphonic acid) with minor modifications. The assay started with the preparation of a stock solution of the radical by reacting equal volumes of ABTS (7.4 μM) and potassium persulfate (2.6 μM) for 12 h with stirring. Standards were prepared by dissolving in methanol to an absorbance of 1.1 ± 0.02 at 734 nm in an EPOCH 2 microplate reader (BIOTEK, Winooski, VT, USA). The antiradical reaction was evaluated for 1 h in the dark at room temperature, and 270 μL of the adjusted working solution of ABTS and 30 μL of *V. microphylla* EO at different concentrations (1, 0.5 and 0.25 mg/mL) were inoculated. The positive and blank controls were Trolox and methanol, respectively. The results were expressed as the SC50 (50% concentration to kill the root) and were calculated according to the appropriate curve fit of the data using GraphPadPrism v. 8.0.1. All measurements were performed in triplicate.

## 5. Conclusions

The aerial parts (stems and leaves) of *V. microphylla* species gave a low yield of essential oil (0.0122% *w*/*w*). The resulting EO was mainly composed of sesquiterpene and monoterpene, the major components of which were α-gurjunene and germacrene D (about 12% each). Enantioselective analysis revealed (+)-α-pinene and (R)-(+)-germacrene as enantiomerically pure compounds. EO exhibited low inhibitory activity against AChE and BuChE enzymes, and a high antioxidant potential was observed for EO, as measured by DPPH and ABTS radical scavenging assays. Furthermore, this genus is of interest for use as a natural antioxidant due to the high activity shown in the present study.

## Figures and Tables

**Table 1:** GC-EIMS and GC-FID analyses of *V. microphylla* Kunth.

		DB-5ms			HP-INNOwax			
N°	Compounds	LRI ^a^	LRI ^b^	% ± SD	LRI ^a^	LRI ^c^	Ref.	% ± SD
1	Isovaleric acid	831	827	0.64 ± 0.65	1676	1680	[20]	2.58 ± 0.07
2	α-Pinene	938	932	0.27 ± 0.25				
3	1,8-Cineole	1031	1026	1.62 ± 0.15	1205	1206	[21]	1.43 ± 0.81
4	n-Nonanal	1105	1100	0.59 ± 0.05	1391	1395	[22]	0.39 ± 0.01
5	(2*E*)-Decenal	1262	1260	0.93 ± 0.42				
6	(*E*)-Anethole	1285	1282	1.16 ± 0.04	1827	1845	[23]	0.28 ± 0.01
7	*α*-Cubebene	1346	1348	0.48 ± 0.14	1451	1460	[22]	0.32 ± 0.07
8	trans-Piperitol acetate	1346	1343	0.33 ± 0.13				
9	Cyclosativene	1367	1369	2.29 ± 0.20	1470	1490	[24]	2.03 ± 0.53
10	*α*-Copaene	1375	1374	6.76 ± 1.80	1462	1464	[25]	6.91 ± 1.59
11	*β*-Cubebene	1386	1387	0.95 ± 0.73	1531	1549	[26]	1.19 ± 0.97
12	*β*-Elemene	1388	1389	2.83 ± 1.46				
13	Sesquithujene	1402	1405	2.19 ± 0.66				
14	*α*-Gurjunene	1406	1409	11.98 ± 3.68	1513	1520	[27]	12.74 ± 5.90
15	*α*-cis-Bergamotene	1412	1411	3.90 ± 1.16	1558	1557	[22]	3.26 ± 2.14
16	(*E*)-Caryophyllene	1418	1417	7.05 ± 0.43	1586	1593	[22]	7.78 ± 0.97
17	*β*-Copaene	1425	1430	0.43 ± 0.19	1580	1585	[22]	2.47 ± 0.80
18	*α*-trans-Bergamotene	1432	1432	0.69 ± 0.31	1563	1580	[22]	1.92 ± 0.51
19	Seychellene	1446	1444	3.03 ± 0.61	1622		[25]	3.69 ± 2.65
20	Sesquisabinene A				1641	1629	[22]	1.74 ± 0.04
21	*α*-Humulene	1453	1444	2.46 ± 0.47	1657	1661	[22]	1.78 ± 0.28
22	allo-Aromadendrene	1458	1458	4.25 ± 1.59	1632	1631	[22]	2.55 ± 0.31
23	(*Z*)-Cadina-1(6),4-diene	1468	1461	1.39 ± 0.44				
24	(*Z*)-β-Farnesene				1647	1665	[22]	0.81 ± 0.20
25	(*E*)-β-Farnesene				1668	1665	[22]	0.54 ± 0.10
26	Ledene				1676	1686	[22]	0.76 ± 0.01
27	Germacrene D	1480	1480	11.47 ± 1.59	1698	1700	[22]	14.93 ± 3.68
28	*γ*-Muurolene				1680	1681	[22]	0.90 ± 0.27
29	*δ*-Selinene	1488	1492	2.43 ± 0.19	1681	1707	[28]	1.78 ± 0.02
30	Bicyclogermacrene	1494	1500	2.41 ± 1.15	1722	1724	[22]	1.40 ± 0.03
31	Isodaucene	1497	1500	0.67 ± 0.24				
32	Pentadecane	1500	1500	0.83 ± 0.83	1501	1500	[29]	0.57 ± 0.04
33	(*E*,*E*)-*α*-Farnesene	1504	1514	4.78 ± 1.31	1748	1746	[22]	4.13 ± 0.51
34	*δ*-Amorphene	1508	1511	0.26 ± 0.06	1716	1704	[25]	1.05 ± 0.01
35	*γ*-Cadinene	1517	1513	2.4 ± 0.40	1750	1748	[22]	3.60 ± 1.29
36	ar-Curcumene				1771	1771	[27]	0.71 ± 0.05
37	Valeric acid				1798	1780	[30]	0.45 ± 0.41
38	(*E*)-Nerolidol	1561	1561	0.56 ± 0.25	2046	2047	[21]	0.39 ± 0.20
39	(*Z*)-calamenene				1814	1808	[22]	0.34 ± 0.01
40	Palustrol	1565	1567	0.46 ± 0.26	1905	1915	[27]	0.31 ± 0.02
41	Spathulenol	1574	1577	0.47 ± 031	2118	2103	[22]	0.32 ± 0.11
42	Caryophyllene oxide	1577	1582	0.43 ± 0.11	1954	1940	[22]	0.30± 0.01
43	Globulol	1582	1590	0.91 ± 0.17	2061	2051	[22]	0.78 ± 0
44	Viridiflorol	1590	1592	0.85 ± 0.41				
45	Ledol	1600	1602	0.50 ± 0.02	2008	2017	[27]	0.88 ± 0.02
46	Hexadecanal				2132	2119	[22]	0.86 ± 0.02
47	*α*-Cadinol	1653	1652	0.45 ± 0.20	2222	2218	[22]	0.09± 0.01
48	Valerianol	1663	1656	0.45 ± 0.14	2243	2230	[31]	0.43 ± 0.05
49	n-Tetradecanol	1677	1671	0.70 ± 0.33				
50	n-Heptadecane	1701	1700	0.64 ± 0.08	1700	1700	[32]	2.09± 0.03
51	Octadecane	1800	1800	0.14 ± 0.08	1800	1800	[26]	0.33 ± 0.03
52	(2*E*,6*E*)-Farnesyl acetate	1845	1845	0.65 ± 0.21				
53	2-Pentadecanone, 6,10,14-trimethyl-	1850	1847	0.42 ± 0.26				
54	n-Nonadecane	1901	1900	0.25 ± 0.02				
55	(*E*,*Z*)-Geranyl linalool	1984	1987	0.65 ± 0.19				
56	1-Eicosene	1996	1987	1.11 ± 0.86				
57	Octadecanal				2345	2345	[33]	0.60 ± 0
58	(*E*,*E*)-Geranyl linalool	2025	2026	1.62 ± 0.04				
59	n-Octadecanol	2087	2077	2.01 ± 0.16				
60	n-Heneicosane	2101	2100	0.34 ± 0.11				
61	n-Tricosane	2302	2300	2.95 ± 1.17	2302	2300	[22]	3.99± 1.32
62	n-Pentacosane				2500	2500	[22]	1.91 ± 0.79
Oxygenated monoterpenes				2.77				1.71
Monoterpene hydrocarbons				0.27				0
Oxygenated sesquiterpenes				5.07				3.49
Sesquiterpene hydrocarbons				75.15				79.31
Aliphatic hydrocarbons				6.27				8.89
Fatty acids				0.64				3.03
Alcohol				2.71				0
Others				5.20				1.85
Total identified oil constituents (%)				98.08				98.29

^a^ Calculated linear retention index on a DB-5MS capillary column; ^b^ Linear retention indices on a DB-5MS column from reference [34]; ^c^ Linear retention indices on a HP-INNOWax capillary column; dLinear retention indices on a HP-INNOWax column from references (Ref.); Relative abundance in percentage (%), expressed as mean ± SD (standard deviation).

**Table 2:** Enantioselective analysis of *V. microphylla* essential oil.

Enantiomer	LRI ^a^	LRI ^b^	Ref.	Enantiomeric Distribution (%)	ee (%)
(+)-*α*-Pinene	931	932	[35]	100	100
(*R*)-(+)-Germacrene	1436	1474	[36]	100	

^a^ Calculated linear retention index; ^b^ Linear retention index from reference; ee (%), percentage of excess enantiomeric.

**Table 3:** Cholinesterase inhibitory activity of *V. microphylla* essential oil.

Sample	AChE, IC_50_ ± SD (µg/mL)	BuChE, IC_50_ ± SD (µg/mL)
*V. microphylla*	>250	>250
Donepezil	0.04 ± 0.01	3.60 ± 0.20

IC_50_; half maximal inhibitory concentration.

**Table 4:** Antioxidant activity of *V. microphylla* essential oil.

Sample	ABTS	DPPH
	SC_50_ (µg/mL)	
*V. microphylla*	41.82 ± 1.62	89.60 ± 1.31
Trolox	23.27 ± 1.05	29.99 ± 1.04

SC_50_; Radical scavenging capacity at 50%.

## Data Availability

Not applicable.
